# Protective Potential of a Botanical-Based Supplement Ingredient against the Impact of Environmental Pollution on Cutaneous and Cardiopulmonary Systems: Preclinical Study

**DOI:** 10.3390/cimb46020099

**Published:** 2024-02-15

**Authors:** Laurent Peno-Mazzarino, Nikita Radionov, Marián Merino, Sonia González, José L. Mullor, Jonathan Jones, Nuria Caturla

**Affiliations:** 1 Laboratoire BIO-EC, Chemin de Saulxier 1, 91160 Longjumeau, France; l.peno-mazzarino@bio-ec.fr (L.P.-M.); n.radionov@bio-ec.fr (N.R.); 2 Bionos Biotech, S.L. Biopolo La Fe, Av. Fernando Abril Martorell, 106, 46026 Valencia, Spain; mmerino@bionos.es (M.M.); sgonzalez@bionos.es (S.G.); jlmullor@bionos.es (J.L.M.); 3 Monteloeder SA, Miguel Servet 16, 03203 Elche, Spain; jonathanjones@monteloeder.com

**Keywords:** air pollution, skin damage, plant polyphenols, nutraceutical, skin explants, human keratinocytes, antioxidants, pulmonary fibroblasts, endothelial cells, medaka embryos

## Abstract

Air pollution is a growing threat to human health. Airborne pollution effects on respiratory, cardiovascular and skin health are well-established. The main mechanisms of air-pollution-induced health effects involve oxidative stress and inflammation. The present study evaluates the potential of a polyphenol-enriched food supplement ingredient comprising *Lippia citriodora*, *Olea europaea*, *Rosmarinus officinalis*, and *Sophora japonica* extracts in mitigating the adverse effects of environmental pollution on skin and cardiopulmonary systems. Both in vitro and ex vivo studies were used to assess the blend’s effects against pollution-induced damage. In these studies, the botanical blend was found to reduce lipid peroxidation, inflammation (by reducing IL-1α), and metabolic alterations (by regulating MT-1H, AhR, and Nrf2 expression) in human skin explants exposed to a mixture of pollutants. Similar results were also observed in keratinocytes exposed to urban dust. Moreover, the ingredient significantly reduced pollutant-induced ROS production in human endothelial cells and lung fibroblasts, while downregulating the expression of apoptotic genes (bcl-2 and bax) in lung fibroblasts. Additionally, the blend counteracted the effect of urban dust on the heart rate in zebrafish embryos. These results support the potential use of this supplement as an adjuvant method to reduce the impact of environmental pollution on the skin, lungs, and cardiovascular tissues.

## 1. Introduction

The combined effects of outdoor and household air pollution are considered a significant threat to human health and the climate. Rapid urbanization and increased energy consumption worldwide have exposed the human body to increased quantities of ambient air pollution, i.e., particulate matter (PM) and its associated components such as polycyclic aromatic hydrocarbons (PAHs), persistent organic pollutants (POPs), and heavy metals. In fact, according to WHO data, almost all the global population (99%) breathe air that exceeds the new WHO guideline limits. The WHO estimates that around 7 million people die every year from exposure to polluted air, leading to diseases such as stroke, heart disease, lung cancer, chronic obstructive pulmonary diseases and respiratory infections, including pneumonia [1,2].

PM, including UFP (<100 nm), PM2.5 (<2.5 μm) and PM10 (2.5–10 μm), originates from both natural and artificial sources such as industrial emissions and vehicle exhausts. These particles exert a profound impact on human health. The most relevant particles are UPF and PM2.5 due to their small molecular diameter and large surface, which mean they can penetrate the deeper respiratory tract and cross the lung capillary network, leading to severe damage in many different tissues and organs. Organs most susceptible to pollution include the cardiovascular system, lungs and skin.

Despite the protective role of human skin against pollution, prolonged and repetitive exposure results in accelerated skin aging [3,4], inflammatory or allergic skin conditions such as atopic dermatitis and eczema [5], psoriasis [6,7], uneven skin pigmentation [8,9], and acne [10,11]. The most deleterious consequence of over-exposure to pollution is skin cancer [12,13]. Ultraviolet radiation (UVR) and environmental air pollutants challenge the skin’s protective ability, with pollutants entering through various routes, including absorption into subcutaneous tissue, hair follicles, and sweat/sebaceous glands [14,15]. Routes by which pollutants enter the skin depend on the skin’s integrity as well as the type and nature of these pollutants. PM, especially of small diameter (PM2.5, UFP), is capable of reaching the dermis via the blood circulation [16,17,18]. Since the protective ability of the skin is limited, problems arise when abnormal exposure to environmental stress exceeds its normal defensive position, leading to the development of various skin diseases [14,15,19].

Pollutants affect the skin on several levels: the skin barrier and skin microflora are altered; there is an increase in oxidative stress (higher ROS production, peroxidation, skin antioxidant depletion, etc.); activation occurs of the aryl hydrocarbon receptor (AhR), which mediates the toxic effects of pollutants; there is an increase in the inflammatory response; and, finally, an increase occurs in matrix metalloproteinase (MMP) activation, resulting in collagen degradation [20].

The respiratory system, comprising the lungs and respiratory pathways, is another organ significantly affected by air pollutants. Since the respiratory system serves as the primary pathway for environmental pollution to enter the human body, the impact of pollutants there is substantial [21]. Long-term exposure to air pollution has been shown to increase the risk of lung diseases such as chronic obstructive pulmonary disease (COPD) and asthma, while short-term exposure can cause airway inflammation, hyperreactivity, decreased pulmonary function, susceptibility to microbial infection, and exacerbation of existing lung diseases [22,23]. Oxidative stress and inflammation are relevant processes triggered by pollutants, with inhaled particles increasing reactive oxygen species production [24,25,26]. Specifically, the oxidative gases such as ozone and NO_2_ enter the lungs, inducing oxidative stress and the inflammatory response [22]. 

Additionally, long-term exposure to air pollution can directly contribute to the occurrence of cardiovascular incidents in part by promoting systemic oxidative stress, inflammation, and autonomic nervous system imbalance. Air pollutants have been linked with endothelial dysfunction and vasoconstriction, increased blood pressure (BP), prothrombotic and coagulant changes, arrhythmias, and eventually atherosclerosis [27,28,29]. Various studies have demonstrated a correlation between elevated levels of air pollutants and changes in heart rate parameters [30,31]. The PM_2.5_ and chemical components present in polluted air can trigger autonomic nervous system responses, leading to alterations in heart rate variability and potentially contributing to cardiovascular complications in susceptible individuals [28,31]. 

Given the prominent role of oxidative stress and inflammation in environmental pollution damage, pharmacological approaches to prevent or reverse the effects of air pollution have centered on compounds with antioxidant properties [32,33,34]. It is also well-established that incorporating dietary antioxidant supplements and/or increasing the intake of fruits and vegetables may help to reduce or protect from the effects of different pollutants [25,35,36]. Recent in vitro and in vivo studies, along with clinical trial interventions, underscore the beneficial effects of plant phenolic compounds in mitigating air-pollution-induced damage to the cardiopulmonary [37,38,39] and skin systems [40,41,42]. Beyond their highly studied antioxidant and anti-inflammatory effects, recent research has highlighted the modulatory capacity of plant-derived compounds on several cellular pathways of interest. Specifically, certain phenolic compounds have been proven to modulate AhR or Nrf2 activity in response to environmental pollutants [43,44]. 

Ideally, studies suggest that consuming a diverse range of antioxidants may be more effective than a high dosage of a single antioxidant, implying potential complementary or synergistic effects [45,46,47]. Therefore, food supplements containing various phenolic compounds from plants with antioxidant and anti-inflammatory properties could be an effective way to prevent the harmful effects of environmental pollution.

Also, in a double-blind placebo-controlled clinical study, we demonstrated the antioxidant and skin anti-aging effects of a commercially polyphenol-enriched food supplement ingredient (trademark family Zeropollution^®^, ZP) comprising *Lippia citriodora*, *Olea europaea*, *Rosmarinus officinalis*, and *Sophora japonica*, in a population exposed to high levels of environmental pollution, particularly PM10 and PM2.5 [42]. Several different scientifically published articles have proven the antioxidant and anti-inflammatory properties of the phenolic compounds present in the above-mentioned botanical blend: hydroxytyrosol, verbascoside, oleuropein, rosemary diterpenes, and quercetin [48,49,50,51,52,53]. In this vein, we hypothesize that such properties attributed to the botanical sources and their polyphenolic compounds could be beneficial for reversing important cellular and molecular changes suffered in different tissues exposed to environmental pollution.

Given the above considerations, the main objective of this study was to evaluate the potential effect of the plant-based supplement ingredient (ZP) in preventing the adverse effects of urban pollution and elucidate its primary mechanism of action in the skin. To achieve this, we gathered results in ex vivo (human skin explants) and in vitro (human keratinocytes) models. Additionally, the possible pollutant-protective efficacy of ZP on the cardiopulmonary system was studied in vitro in human pulmonary fibroblasts, human endothelial cells and medaka fish (*Oryzias latipes*) embryos. 

## 2. Materials and Methods

### 2.1. Experimental Product

The test item was a patented (WO/2019/211501), commercially available food supplement ingredient (trademark family Zeropollution^®^) supplied by Monteloeder S.L., Miguel Servet 16, Elche, Alicante, Spain. The ingredient is a blend of four polyphenolic botanical extracts: *Rosmarinus officinalis* leaf extract standardized in diterpenes, *Olea europaea* leaf extract standardized in oleuropein and hydroxytyrosol, *Lippia citriodora* leaf extract standardized in verbascoside and *Sophora japonica* extract standardized in quercetin. The four materials were extracted with water, ethanol or a mixture of both, and dried. Prior to blending, the four herbal extracts were standardized to ensure a consistent and specific content of active compounds. The final blend was sieved to obtain a uniform particle size. More detail can be seen in Figure S1 of the supplementary section and in the above-mentioned patent. In total, *w*/*w* this blend comprises a minimum content of the following phenols: diterpenes (mainly sum of carnosic acid and carnosol) 4.5%; oleuropein 4.5%; hydroxytyrosol 1.5%; verbascoside 6.8%; and flavones such as quercetin minimum 3.7%. These main compounds were identified and quantified by HPLC-DAD analysis, comparing the retention times and UV spectra of the peaks in samples with those of authentic standards as previously described [42]. In addition, other phenolic compounds identified by HPLC and HPLC-MS analysis included phenolic acids like chlorogenic acid, caffeic acid and rosmarinic acid, phenylethanoids such as tyrosol and isoverbascoside and different flavones, mainly apigenin derivatives, luteolin derivatives and rutin. More details of the method used and active components identified can be seen in Figure S2 and Table S1 of the supplementary section. 

Two different batches of the ingredient were employed in this study. The batch employed for ex vivo evaluation contained the following percentage of active components: 4.83% diterpenes, 4.71% oleuropein, 1.78% hydroxytyrosol, 7.53% verbascoside, and 4.45% flavones such as quercetin. On the other hand, the batch used for the remaining studies contained 4.69% diterpenes, 4.83% oleuropein, 1.69% hydroxytyrosol, 7.13% verbascoside, and 4.80% flavones such as quercetin. 

In the context of this study, the ingredient is referred to as ZP.

### 2.2. Ex Vivo Assessment of the Anti-Pollution Activity of ZP on Human Living Skin Explants

#### 2.2.1. Skin Explants

Human skin biopsies were obtained from an abdominoplasty of a 56-year-old Caucasian female. The BIO-EC Laboratory is authorized by the Bioethics group of the French Research and Innovation Ministry (registered n° DC-2008-542) to use human skin from surgical waste (authorized since May 2010). The study was performed in accordance with the Declaration of Helsinki and patients gave informed consent for us to use their skin samples.

In total, 19 human skin explants with an average diameter of 12 mm (±1 mm) were prepared and kept in BEM culture medium (BIO-EC’s Explant Medium, Longjumeau, France) in a humidified atmosphere with CO_2_ (5% *v*/*v*) and a temperature of 37 °C. Explants were then divided into 5 test batches and grouped according to treatment plan (Table 1).

#### 2.2.2. Pollutants and Treatment

**Figure 1:**
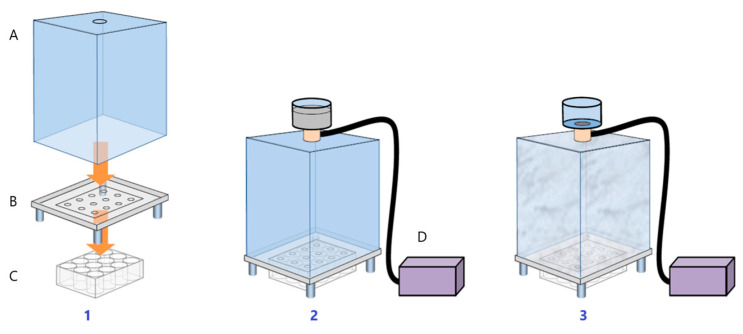
Set-up of PolluBox. 1: The system is composed of a chamber (A) and a base (B) both made of poly (methyl methacrylate) resin. The base (B) contains 12 holes with a diameter of 8 mm, restricting the exposure to the skin explant surface alone. For the exposure to pollutants, skin explants are placed in a classical 12-well cell culture plate (C) with culture medium. 2: The culture plate is then positioned on the base of the Pollubox in order to align skin explants at the levels of the holes of the base. A nebulizer (Aerogen Pro) (D), placed on the top of the chamber, allows us to nebulize the liquid solution containing the pollutants. 3: The generated aerosol precipitates uniformly on the surface of skin explants placed at the base of the Pollubox, avoiding any systemic contamination of the samples.

We established an experimental model wherein living skin explants maintained in culture were exposed to a combination of pollutants. To this end, a mixture of pollutants mimicking urban pollution was formulated based on the French organization ‘Air Parif’ database. To facilitate the controlled nebulization of pollutants onto the cultured human skin explant, we used a chamber called Pollubox^®^ (Figure 1). This model and the pollutants used were previously validated as an efficient tool to study the effects of pollution on skin and evaluate cosmetic product efficacy [54].

The pollutant mix (PM) used was Merck ICP standardized heavy metals (0.0005–0.01 mg/mL) supplemented with benzene, xylene, toluene, and diesel particles (NIST). A detailed description of the composition can be found in the supporting information section (Table S2). According to measurements taken during the development of this model, the quantity of nebulized pollutants deposited on each explant was set at 30 mg, representing 1% of the nebulized quantity.

On day 0, explants were placed in 2 mL of culture medium with or without the test ingredient ZP at 200 µg/mL Controls T0 and T did not receive any treatment. On day 2, the explants of test batches were placed into the PolluBox^®^ system. Test batches P and PZP were then “exposed” to a pollutant mix by vaporization for 90 min, whereas explants of test batches T and ZP were not exposed. After exposure, all explants were returned to an incubator under standard culture conditions for 24 h.

#### 2.2.3. Sampling and Histological Processing

Culture medium from all batches was collected and stored at −80 °C for MDA and IL1α assays. Explants were collected on day 3 except for the test batch T0 that was collected on day 0. After fixation for 24 h in buffered formalin, samples were dehydrated and impregnated in paraffin. Samples were paraffin embedded and 5 µm thick sections were obtained using a Leica Minot-type microtome and mounted on Superfrost^®^ histological glass slides. Microscopy observations were obtained using a Leica DMLB or Olympus BX43 microscope. Images were digitized using a numeric DP72 Olympus camera with CellD storing software version 5174 . Cell viability was obtained on paraffin sections treated with Masson’s trichrome staining and Goldner’s variant [55] and assessed via observations of both epidermal and dermal structures.

#### 2.2.4. Immuno-Histochemistry

All explants were stained for the following: AHR (aryl hydrocarbon receptor), MT-1H (metallothionein) and Nrf2 (oxidative stress transcription factor). Each immunostaining was conducted on formol fixed paraffin-embedded sections with monoclonal antibody (anti-AHR, ThermoScientific, Waltham, MA, USA, MA1-514, clone RPT1; anti-MT1H Dako, M0639, clone E9; anti-Nrf2, abcam ab76026, clone EP1809Y), and biotin-conjugated secondary antibody. Staining was performed using the HRP–avidin/biotin complex (Vector Vectastain RTU Universal) and a violet substrate of peroxidase (VIP, Vector laboratories Inc., Newark, NJ, USA, SK4600). Immunostaining was performed using an automated slide processing system (Autostainer, Dako, Santa Clara, CA, USA) and assessed by microscopic observations.

#### 2.2.5. Biochemical Assays—MDA and IL-1α

The MDA (malondialdehyde) assay for lipid peroxidation was performed with an enhanced method of the thiobarbituric acid reactive substance assay (TBAR). To enhance the specificity of the assay, MDA was extracted using liquid/liquid extraction with butanol. The MDA in butanol was then measured by spectrofluorometry (excitation: 515 nm, emission: 550 nm) using a Tecan Infinite M200 Pro micro-plate reader. On day 3, MDA present in the culture medium was measured. MDA was analyzed in culture mediums of all explants (*n* = 4) collected on day 3.

The IL-1α cytokine assay was performed using a human IL1-α ELISA kit (Cayman Chemicals, Ann Arbor, MI, USA). Culture medium and the IL-1α standard were incubated with an acetylcholinesterase (AChE): Fab’ conjugate for binding IL-1α in wells containing immobilized IL-1α antibody, for 12 h at 4 °C. After well plate washing, the reaction was observed for 40 min using a solution containing AChE substrate. Absorbance at 412 nm was measured using an M200Pro Tecan micro-plate reader with Magellan 7 software version 2. IL1-α was analyzed in culture mediums of all explants (*n* = 4) collected on day 3. 

#### 2.2.6. Image Analysis and Statistics

Image analysis was carried out on all immunostaining images using Olympus CellD software version 5174. Once the area of interest was selected (epidermis), the surface occupied by the staining was then measured and expressed as a percentage of the surface.

Data from image analysis or biochemical assays were subjected to statistical analysis according to Student’s *t*-test with GraphPad Prism software version 9.0.0. Statistical significance was set at *p* < 0.05 (95% confidence).

### 2.3. In Vitro Analysis of the Anti-Pollution Capacity of ZP in Human Keratinocytes

#### 2.3.1. Cell Culture Conditions

Confluent HaCaT cells cultured on a 75 cm^2^ Nunc™ EasYFlask™ (Thermo Fisher Scientific, Waltham, MA, USA) were detached by incubation with Trypsin-EDTA 0.5% without phenol red (Gibco, Waltham, MA, USA) over 5 min at 37 °C and 5% CO_2_. Trypsin was then inactivated by adding 5 volumes of DMEM 1 g/L glucose (Gibco, Waltham, MA, USA supplemented with 10% FBS (Gibco, Waltham, MA, USA) (hereafter D10 medium) and mixing thoroughly. The obtained cell suspension was mixed 1:1 with trypan blue 0.4% and incubated for 30 s at room temperature. Cell counting of the trypan-blue-diluted cell suspension was performed in a Countess II Automated Cell Counter (Thermo Fisher Scientific, Waltham, MA, USA). Accordingly, cells were conveniently diluted in D10 medium to a final density of 10^4^ cells/well in a Nunc™ MicroWell™ 96-Well (Thermo Fisher Scientific , Waltham, MA, USA) for antioxidant assessment and in 12-well plates (Thermo Fisher Scientificat, Waltham, MA, USA) at a density of 250,000 cells/well for ELISA quantification. Then, cells were incubated overnight at 37 °C and 5% CO_2_. Afterwards, 10 medium was removed and replaced by DMEM 1 g/L glucose supplemented with 0.5% FBS (hereafter D0.5) to prevent any possible interaction of the proteins from FBS with UD components or the tested products, while retaining basal growth and metabolism of the cultured cells. The UD used comprised particles of atmospheric matter collected from air filters in the urban area of the city of Valencia. Details of the UD used can be found in the supplementary section (Table S3).

#### 2.3.2. Antioxidant Assessment against UD-Induced Oxidative Stress

HaCaT cells were cultured for 24 h in the presence (or absence) of 400 µg/mL UD and either 0.01% or 0.005% ZP. The UD suspension was sonicated for 30 min to avoid aggregation of particles before adding it to the cell medium. After 24 h of incubation, the culture medium of all wells was replaced with PBS and ROS master mix (Sigma-Aldrich, Darmstadt, Germany) in all culture wells, including the 2 blank controls (wells without cells for basal signal determination), and that was left for 1 h. Non-treated controls with cells were incubated at 37 °C during this time in the dark. Immediately after the treatment, ROS were measured in all samples. The intracellular ROS reacted with a fluorogenic sensor localized in the cytoplasm, resulting in a fluorometric product in an amount proportional to the amount of ROS present. Fluorescence quantification was measured by spectrofluorometry (excitation: 490 nm, emission: 525 nm) using a Multiskan EX Primary EIA plate reader (Thermo Scientific). 

In parallel, cell viability was quantified through MTT assay (Nr.17, ECVAM) [56], which allowed us to normalize ROS levels to the number of live cells to avoid false positives due to UD-induced cytotoxicity. One biological replicate with six technical replicates for each condition, covering all the conditions, were used.

The blank control mean was subtracted from all sample data, and, afterwards, the data were corrected to the mean of the cell viability assay performed in parallel. Finally, data were normalized to the control + UD, represented as mean ± SEM, and analyzed statistically, comparing UD-treated samples vs. UD control. The test applied for the analysis was Student’s *t*-test. Statistical significance was set at *p* < 0.05 (95% confidence). 

#### 2.3.3. Antioxidant Assessment against UD-Plus-UVA-Induced Oxidative Stress (Photopollution Model)

HaCaT cells were cultured for 24 h in the presence (or absence) of 400 µg/mL UD and 0.001% and 0.005% ZP. Following incubation, HaCaT sample medium was replaced with a ROS detection kit (Sigma-Aldrich, Darmstadt, Germany), and samples were irradiated for 25 min with UVA (6.9 J/cm^2^: Luzchem irradiator LZC-420). Non-irradiated and non-treated controls were maintained in the dark at 37 °C during this period. Reactive ROS concentrations in the test samples were measured 2 h later by spectrophotometry, as described above. Cell viability analysis by MTT was also performed to assess UD and UD + UVA-induced cell mortality, normalizing ROS levels to the quantity of living cells in each specific condition. 

For the analysis of results, the blank control was subtracted from the sample data. Then, relative ROS levels with respect to the cell survival average were calculated from each condition and normalized with respect to control + UD + UVA. These values were represented as mean ± SEM, analyzed statistically (Students *t*-test) comparing UD + UVA-treated samples versus UD + UVA control samples. Statistical significance was set at *p* < 0.05 (95% confidence).

#### 2.3.4. Anti-Inflammatory Assessment against UD-Induced IL-6 and IL-1α Production

Human HaCaT keratinocytes were cultured over 24 h in the presence of ZP at 2 concentrations, 0.0025% and 0.001%. The inflammatory response was induced by using UD at 400 μg/mL during the same period. After the incubation period, cell culture supernatants were harvested and centrifuged at 350× *g* for 5 min to separate UD particles from soluble components. The UD-depleted supernatants were then used to measure the levels of secreted IL-6 and IL-1α using the Human IL-6 and IL-1a ELISA Kit (ab178013 and ab178008, Abcam). Then, the assay was performed as per the manufacturer’s instructions. The obtained concentration values of IL-6 and IL-1α were corrected versus absorbance values obtained from an MTT assay performed in parallel in the attached cells, indicative of cell viability after the treatment. A total of 4 technical replicates per concentration were performed for the ELISA analysis. The MTT assay was set with 8 replicates per condition. The final values were represented as mean ± SEM, analyzed statistically (Students *t*-test) comparing UD-treated samples versus UD control samples. Statistical significance was set at *p* < 0.05 (95% confidence).

#### 2.3.5. Evaluation of AhR, Nrf2, and CYP1A1 Protein Levels

Human HaCaT keratinocytes were cultured for 24 h in the presence of ZP at 2 concentrations, 0.0025% and 0.001%, and UD at 400 μg/mL. After the incubation period, cell lysates were processed for AhR, Nrf2, and CYP1A1 measurements, as per manufacturer’s ELISA kit instructions (BHLHE76, SEL947Hu and SED295Hu, Cloud-Clone Corp., Park Row, TX, USA). A total of 4 technical replicates per concentration were performed for the ELISA analysis. A total of 8 replicates per condition was set for the MTT assay. The final values were represented as mean ± SEM, analyzed statistically (Students *t*-test) comparing UD-treated samples versus UD control samples. Statistical significance was set at *p* < 0.05 (95% confidence).

### 2.4. In Vitro Analysis of the Anti-Pollution Capacity of ZP in Human Pulmonary Fibroblasts

#### 2.4.1. Cell Culture Conditions

Confluent human pulmonary fibroblasts (HPFs) (Promocell, Heidelberg, Germany) cultured on a 75 cm^2^ Nunc™ EasYFlask™ (Thermo Fisher Scientific, Waltham, MA, USA) were detached by incubation with trypsin–EDTA 0.5% without phenol red (Gibco) over 5 min at 37 °C and 5% CO_2_. Trypsin was then inactivated by adding 5 volumes of D10 medium and mixing thoroughly. The obtained cell suspension was mixed 1:1 with trypan blue 0.4% and incubated for 30 s at room temperature. Cell counting of the trypan-blue-diluted cell suspension was performed in a Countess II Automated Cell Counter (Thermo Fisher Scientific, Waltham, MA, USA). Accordingly, cells were conveniently diluted in D10 medium to a final density 10^4^ cells/well in Nunc™ MicroWell™ 96-Well (Thermo Fisher Scientific, Waltham, MA, USA) for antioxidant assessment and in 6-well plates (Thermo Fisher Scientificat, Waltham, MA, USA) at a density of 300,000 cells/well for RNA extraction. Then, cells were incubated overnight at 37 °C and 5% CO_2_. Afterwards, D10 medium was removed and replaced with D0.5. Details of the UD used in the studies can be found in the supplementary section (Table S2).

#### 2.4.2. Antioxidant Assessment against UD Induced Oxidative Stress

HPF cells were cultured for 24 h in the presence (or absence) of 400 µg/mL UD and in the presence of 0.0001% or 0.0005% ZP. The UD suspension was sonicated for 30 min before adding it to the cell medium. After 24 h of incubation, the culture medium in all wells was replaced by PBS and ROS master mix (Sigma-Aldrich, Darmstadt, Germany) for 1 h, including in the 2 blank controls (wells without cells for basal signal determination). Non-treated controls cells were incubated at 26 °C during this time in the dark. Immediately after the treatment, ROS was determined by fluorescence quantification (λ ex: 490 nm, λ em: 525 nm) using a Multiskan EX Primary EIA plate reader (Thermo Scientific).

In parallel, cell viability was quantified through MTT assay, in order to normalize ROS levels to the number of live cells to avoid false positive results due to UD-induced cytotoxicity. We used one biological replicate with six technical replicates for all the conditions. For statistical analysis of the results, the blank control mean was subtracted from all sample data, and afterwards, all data were corrected to the mean of the cell viability assay performed in parallel. Finally, data were normalized to control + UD, represented as mean ± SEM, and analyzed statistically comparing UD-treated samples vs. UD control. The test applied for the analysis was Student’s *t*-test. Statistical significance was set at *p* < 0.05 (95% confidence).

#### 2.4.3. Study of mRNA Expression of bcl-2 and Bax Genes by RT-qPCR

HPF cells were cultured for 24 h in culture medium supplemented with ZP (0.0001% and 0.0005%) and with UD at 400 µg/mL. A control without any treatment and control without product treatment but with UD treatment were included in the assay. After 24 h of incubation, the medium was removed and cells were washed with PBS. Cells were then collected in lysis buffer to proceed with RNA extraction. Total RNA was extracted from cell aliquots using an RNeasy mini kit (Qiagen, Venlo, The Netherlands). And treated with DNAse-I (Qiagen) to remove any contamination from genomic DNA. RNA quality and quantity were checked in a Nano-Drop spectrophotometer (Applied Byosystem, Foster City, CA, USA), and 500 ng of total RNA was used to synthesize cDNA. The suitability of each primer pair used in this study for RT-qPCR, ACT (internal control housekeeping gene), BAX2 and FAS1 was previously evaluated to determine melting curves, efficiency of amplification and specificity of the primers. Quantitative PCR was performed in a real-time PCR machine (QuantStudio 5 Applied Biosystem). 

To perform raw data analysis, the Pfaffl method [57] was used to calculate the gene relative expression ratio to ACT. The mathematical model of the relative expression ratio in RT-PCR was calculated as follows: $ratio=\frac{{\left(E\mathrm{target}\right)}^{A\Delta CPtarget(control-sample)}}{{\left(E\mathrm{ref}\right)}^{A\Delta CPref(control-sample)}},$ where *E_target_* is the real-time PCR efficiency of the target gene transcript; *E_ref_* is the real-time PCR efficiency of a reference gene transcript; Δ*CP_target_* is the difference between the CP deviation of the control and the sample of the target gene transcript; and Δ*CP_ref_* is the difference between CP deviation and the sample of the reference gene transcript. 

A total of four technical replicates per condition were performed. Statistical analysis to determine significant changes was performed using an unpaired Student’s *t*-test. For all data, a level of 5% or less (*p* < 0.05) was taken as statistically significant.

### 2.5. In Vitro Antioxidant Assessment against UD-Induced Oxidative Stress in Human Endothelial Cells (HUVECs)

Human umbilical vein endothelial cells (HUVECs) (Promocell, Heidelberg, Germany) cultured on a 75 cm^2^ Nunc™ EasYFlask™ (Thermo Fisher Scientific, Waltham, MA, USA) were detached by incubation with trypsin–EDTA 0.5% without phenol red (Gibco) over 5 min at 37 °C and 5% CO_2_. Trypsin was then inactivated by adding 5 volumes of Endothelial Cell Growth Medium 2 (Promocell, Heidelberg, Germany) and mixing thoroughly. The obtained cell suspension was mixed 1:1 with trypan blue 0.4% and incubated for 30 s at room temperature. Cell counting of the trypan-blue-diluted cell suspension was performed in a Countess II Automated Cell Counter. Accordingly, cells were conveniently diluted in endothelial cell growth medium to a final density of 10^4^ cells/well in Nunc™ MicroWell™ 96-Well. 24 h later, the culture medium was removed and substituted for new culture medium D 0.5 supplied with 400 μg/mL of UD and ZP at 0.0005% and 0.001%. After 24 h of incubation, ROS were measured as described above. Details of the UD used can be found in the supplementary section (Table S2). A total of six technical replicates for all the conditions were used. For statistical analysis of results, the blank control mean was subtracted from all sample data, and all data were corrected to the mean of the cell viability assay performed in parallel. Finally, data were normalized to control + UD, represented as mean ± SEM, and analyzed statistically comparing UD-treated samples vs. UD control. The test applied for the analysis was Student’s *t*-test. Statistical significance was set at *p* < 0.05 (95% confidence).

### 2.6. Heartbeat Quantification in Medaka Embryos Exposed to Urban Dust

The medaka fish (*Oryzias latipes*) is scientifically demonstrated to be a suitable model for performing screenings in biomedicine, pharmacology and cosmetics [58,59]. Like in the HetCam assay with chicken embryos, assays with medaka embryos are considered in vitro testing and comply with the new EU Regulation 655/2013 [60]. The fish’s rapid developmental process leads to a functional cardiovascular system emerging by around 48 h post fertilization (hpf) [61]. Although the fish heart is two-chambered, its development and function are very similar to the mammalian heart [62]. Remarkably, components of the electrocardiogram of medaka or zebrafish have more similarities to those in humans than rodents, making them ideal models to investigate heart rate and rhythm phenotypes and leverage these properties for drug screens [63,64]. 

For all the above reasons, in this study, we assess the capacity of ZP in counteracting the UD impact on the heart rate of medaka embryos. Medaka fish were donated by the Principe Felipe Research Center and maintained in the animal facilities of Hospital La Fe in Valencia. Adult medaka (*Oryzias latipes*) CAB strain animals were maintained in recirculating water aquaria on a 14 h light/10 h dark daily cycle at 28 °C. Fish embryos were collected by natural spawning in Yamamoto buffer (NaCl, CaCl_2_, NaHCO_3_ from Sigma-Aldrich, KCl from USB) and at 22–23 developmental stage (3 days old) were dispensed individually in a 96-well plate and treated with ZP at two different concentrations (0.005% and 0.01%) and with UD at 250 μg/mL. Controls without any treatment and controls without product but with UD were included in the assay. After 48 h of product and UD exposure (eleutheroembryos at 30–31 developmental stage), the heart rate was quantified by recording the embryos for 30 s using a stereoscopic microscope (Leica, Weltzar, Germany). Details of the UD used can be found in the supplementary section (Table S2). Five technical replicates of five eleutheroembryos/condition were used. All data were statistically analyzed by an unpaired Student’s *t*-test. Statistical significance was set at *p* < 0.05 (95% confidence). Graphical results were represented as mean ± SEM, analyzed statistically compared to the control + UD samples.

## 3. Results

### 3.1. Ex Vivo Assessment of the Anti-Pollution Activity of ZP on Human Living Skin Explants

#### 3.1.1. Lipid Peroxidation (MDA) and Anti-Inflammatory (IL-1α) Assay Results

Malondialdehyde (MDA) is a low-molecular-weight end-product arising from lipid peroxidation of the cell membranes. The free radicals induced by oxidative stress, e.g., UV, pollution, etc., degrade polyunsaturated lipids and generate hydro-peroxides, resulting in the formation of radical intermediates and aldehydes, particularly MDA [65]. 

Likewise, inflammation is a physiological response that protects the body from various insults, such as physical injury, pathogens, exposure to toxic chemicals, and UV irradiation. Acute inflammation has a widely recognized physiological function, provided that its duration is strictly regulated; however, prolonged inflammation can lead to several diseases. Studies have shown that exposure to PM and heavy metals significantly induced the release of IL-1α in the epidermis [66].

**Figure 2:**
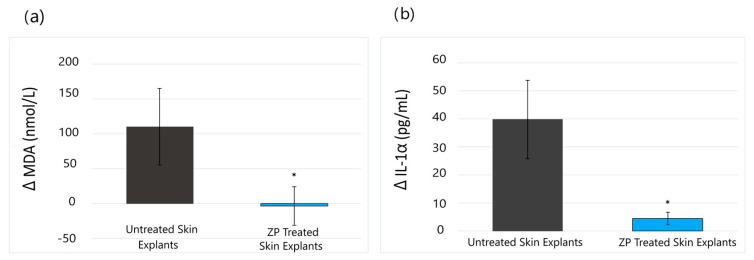
(**a**) Delta (increase) of MDA induced by pollutant mixture for each explant compared to the average of the batch without pollutant mixture. Average delta of the MDA induction in control without treatment (gray bar) and in the presence of ZP (blue bar). (**b**) Delta (increase) of IL-1α induced by pollutant mixture for each explant compared to the average of the batch without pollutant mixture. Average delta of the IL-1α induction in control without treatment (gray bar) and in the presence of ZP (blue bar). Data are expressed as the mean ± SD of 4 independent skin explants. * Statistical significance with *p* < 0.05.

The results of our study revealed that ZP presented a protective effect against lipid peroxidation induced by the pollutant mix on human skin explants. On day 3, a total inhibition of lipid peroxidation (MDA), induced by the pollutant, was achieved with 200 µg/mL of ZP (ΔPZP vs. ΔP) (Figure 2a). On the other hand, ZP also significantly inhibited the inflammatory response and reduced the increase in IL1-α induced by pollutants by 89% (*p* < 0.05) (ΔPZP vs. ΔP) (Figure 2b). 

#### 3.1.2. AhR Expression

The AhR (aryl hydrocarbon receptor) receptor is a transcriptional factor implicated in molecular response following the exposure to several compounds, including aromatic polycyclic hydrocarbons, ozone and plant polyphenols. AhR is expressed by several cell types, including keratinocytes, melanocytes, Langerhans cells and T cells [67], and is considered a valuable marker for measuring changes to skin metabolism under stress. In addition, exposure to substances such as PAHs trapped in PM causes skin aging by activating MMP-1 through the AhR pathway [25,26,27,28]. AhR activation also mediates PM-induced upregulation of COX2 expression and PGE2 production in the skin barrier, causing additional damage [29].

**Figure 3:**
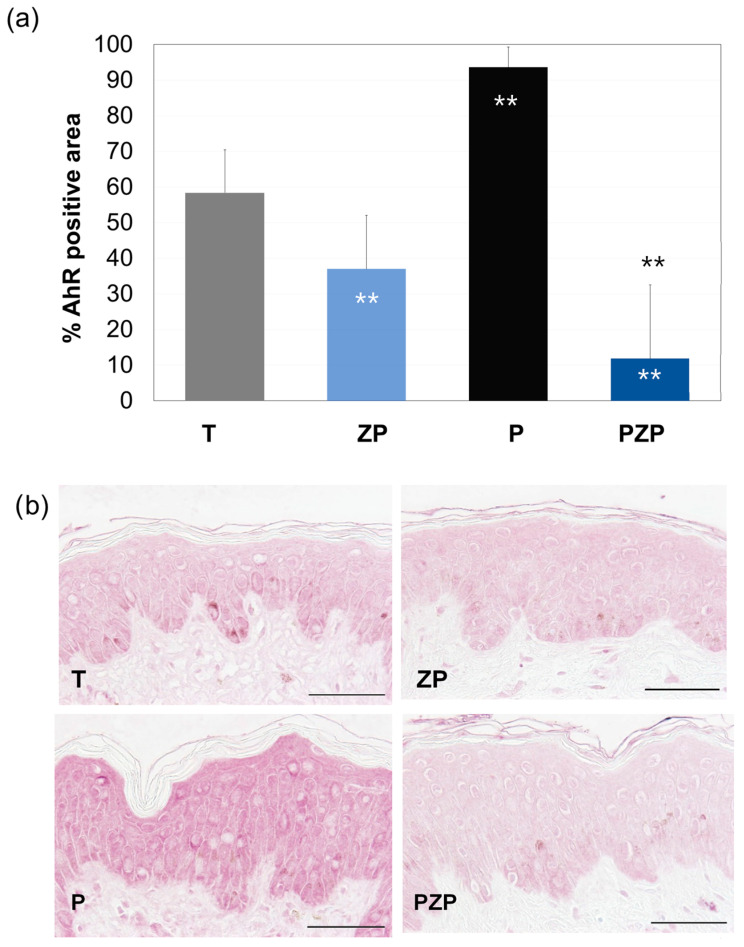
(**a**) Bars represent the surface percentage positive in AhR immunostaining in the living skin explant epidermis on day 3 of different batches. Data are expressed as the mean ± SD of 4 independent skin explants. (**b**) Representative images of AhR staining of skin explants at day 3 of different batches. Scale bars = 50 µm. Batches: untreated control (T), ZP treatment (ZP), pollution treatment (P) and pollution + ZP treatment (PZP). ** Inside the bars represent statistical significance with *p* < 0.01 vs. untreated control (T). ** Above the bars represent statistical significance with *p* < 0.01 vs. pollution treatment (P).

The results of this study are shown in Figure 3. After 3 days, in the untreated control batch (T), 58.4% of the surface of the living epidermis was positive in AhR immunostaining. The addition of ZP caused a 37% decrease (*p* < 0.01) in AhR expression compared to untreated skin explants (T). On the other hand, exposure of the skin explants to pollution significantly increased (60%, *p* < 0.01) AhR expression compared to T. When ZP was added, an 87% decrease (*p* < 0.01) in AhR expression was observed compared to pollutant-exposed skin explants (P) (Figure 3).

#### 3.1.3. Metallothionein (MT-1H) Expression 

Metallothioneins (MTs) are small cysteine-rich proteins that play important roles in metal homeostasis and protection against heavy metal toxicity, DNA damage, and oxidative stress [68]. MT1H can also be induced by inflammatory cytokines, after UV irradiation [69].

**Figure 4:**
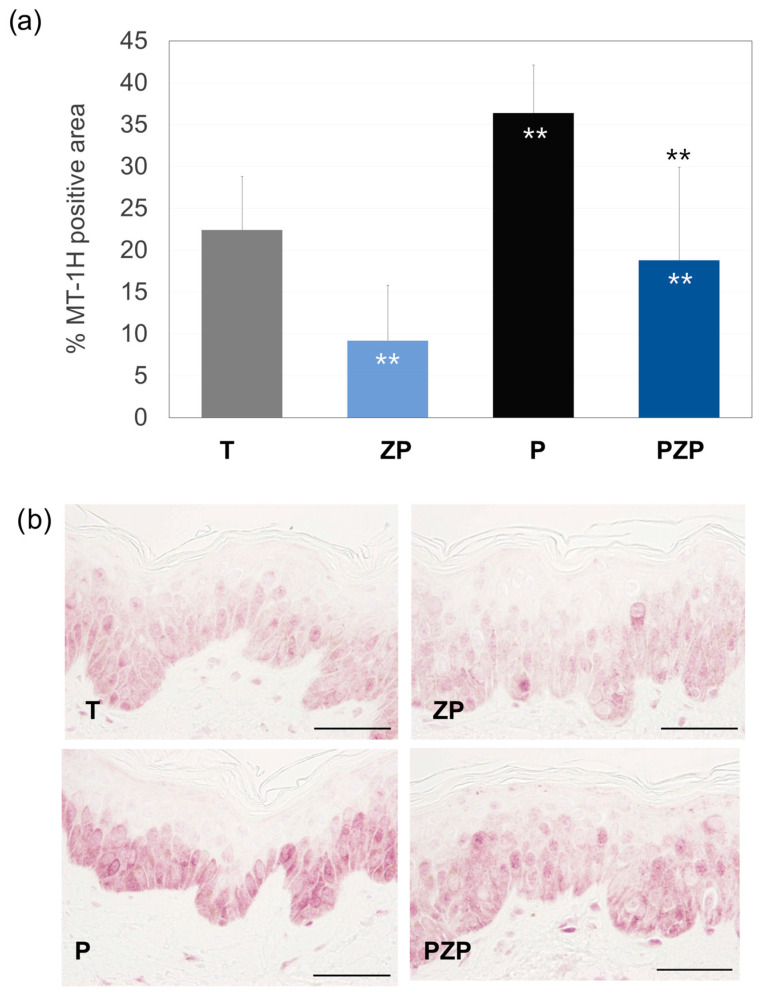
(**a**) Bars represent the surface percentage positive in MT-1H immunostaining in the living skin explant epidermis on day 3 of different batches. Data are expressed as the mean ± SD of 4 independent skin explants. (**b**) Representative images of MT-1H staining of skin explants at day 3 of different batches. Scale bars = 50 µm. Batches: untreated control (T), ZP treatment (ZP), pollution treatment (P) and pollution + ZP treatment (PZP). ** Inside the bars represent statistical significance with *p* < 0.01 vs. untreated control (T). ** Above the bars represent statistical significance with *p* < 0.01 vs. pollution treatment (P).

The results of this study showed that after 3 days, in the untreated control batch (T), 22.4% of the surface of the living epidermis was positive in MT-1H immunostaining. The inclusion of ZP significantly decreased staining by 59% (*p* < 0.01) compared to the T batch. Exposure of the skin to pollution led to a significant increase (63%, *p* < 0.01) in MT-1H expression compared to T. The treatment with ZP, compared to the pollutant skin explant control batch (P), significantly reduced this expression by 48% (*p* < 0.01) (Figure 4).

#### 3.1.4. Nrf2 Expression

The nuclear factor erythroid 2–related factor 2 (Nrf2) is a key transcription factor in the cellular response to oxidative stress and regulates the expression of phase II detoxifying enzymes. Nrf2 plays a key role in preventing PM-induced toxicity by protecting against oxidative damage and inflammation, and it has been demonstrated that exposure to metals present in urban PM can induce the activation and expression of Nrf2/antioxidants/phase II detoxifying enzymes, as part of the protection against oxidative stress [70]. 

**Figure 5:**
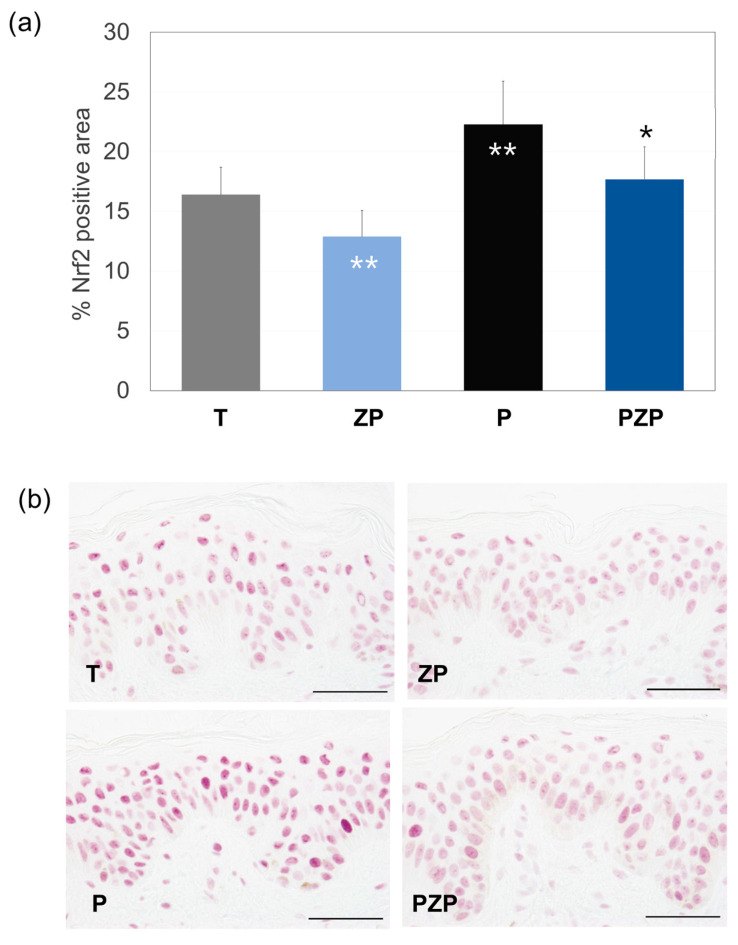
(**a**) Bars represent the surface percentage positive in Nrf2 immunostaining in the living skin explant epidermis on day 3 of different batches. Data are expressed as the mean ± SD of 4 independent skin explants. (**b**) Representative images of Nrf2 staining of skin explants at day 3 of different batches. Scale bars = 50 µm. Batches: untreated control (T), ZP treatment (ZP), pollution treatment (P) and pollution + ZP treatment (PZP). ** Inside the bars represent statistical significance with *p* < 0.01 vs. untreated control (T). * Above the bars represent statistical significance with *p* < 0.05 vs. pollution treatment (P).

The findings of this research are shown in Figure 5. After 3 days, for the untreated control batch, 16.4% of the surface of the living epidermis was positive in Nrf2 immunostaining. The addition of ZP decreased Nrf2 expression by 22% (*p* < 0.01) compared to untreated control skin explants (T). Exposure of the skin to pollution induced a significant increase in Nrf2 expression by 36% (*p* < 0.01) compared to the T batch. The treatment with ZP significantly reduced Nrf2 expression by 21% (*p* < 0.01), compared to the pollutants skin explant control; however, a significant increase was observed when compared to the explants treated with ZP but not exposed to a pollutant (ZP vs. PZP).

### 3.2. In Vitro Analysis of the Anti-Pollution Capacity of ZP in Human Keratinocytes

#### 3.2.1. Antioxidant Assessment against UD-Induced Oxidative Stress

**Figure 6:**
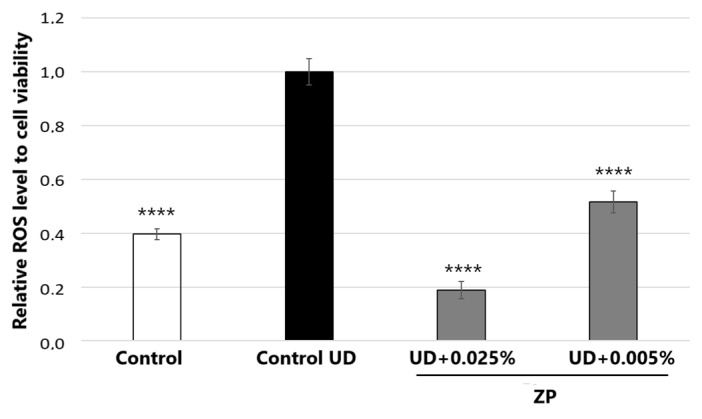
ROS accumulation relative to cell viability and normalized to control UD. Results were obtained after 24 h of treatment in HaCaT cells treated with ZP at 0.005% and 0.025% and simultaneously exposed to UD. Data are expressed as the mean ± SEM of 6 technical replicates per condition and **** represent statistical significance with *p* < 0.0001 compared to the control UD.

Figure 6 depicts the treatment with UD at 400 μg/mL on human keratinocyte cells, which significantly increased ROS levels by 152.2 ± 13.5%, compared to untreated control cells after 24 h of incubation. When cells were also incubated with the product for 24 h, the results indicated that treatment with ZP at 0.005% and 0.025% on HaCaT cells significantly decreased ROS levels by 48.3 ± 6.4% and 81.2 ± 5.8%, respectively, compared to the untreated control + UD.

#### 3.2.2. Antioxidant Assessment against UD-Plus-UVA-Induced Oxidative Stress

**Figure 7:**
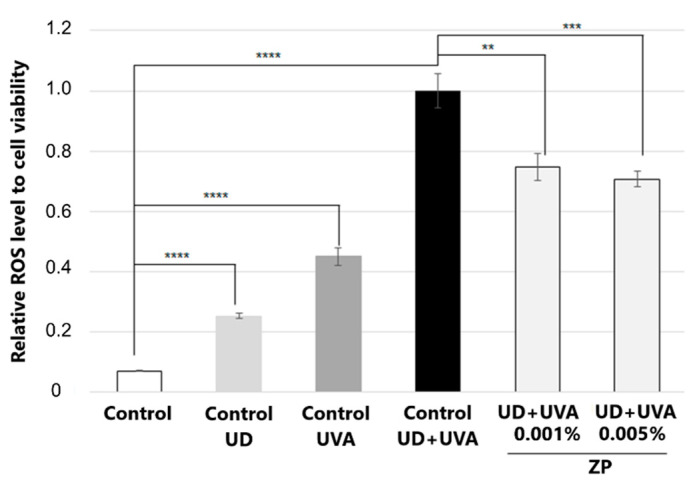
ROS accumulation relative to cell viability and normalized to control UD. Results were obtained in samples untreated and treated with ZP at 0.001% and 0.005%, and UD treatment for 24 h before UVA irradiation for 25 min, on HaCaT cells. Data are presented as the mean ± SEM of 8 technical replicates per condition and ** (*p* < 0.01), *** (*p* < 0.001) and **** (*p* < 0.001) indicate statistical significance.

Exposure to UVA and airborne pollutants simultaneously causes synergistic damage and accelerated extrinsic aging with increased carcinogenesis [18]. In this context, we studied whether ZP could inhibit photo-pollution-induced ROS formation in UD-treated keratinocytes. The results of this study are shown in Figure 7.

When keratinocytes were not exposed to external stressors such as UD and UVA, the addition of ZP at a concentration of 0.005% led to a statistically significant reduction in oxidative stress (ROS) levels, resulting in a 14.1% decrease ± 4.8% (*p* < 0.05) compared to the untreated control group.

In parallel, the results of this study indicated that both UD and UVA increased ROS formation in a significant manner; however, when both stressors were combined, the ROS increase was much higher, doubling that of UVA radiation alone and 13.2 ± 0.8-fold higher than that of the untreated control group (Figure 7). Interestingly, when cells were pre-treated with ZP and then incubated with UD for 24 h followed by 25 min of UVA radiation, the results demonstrated that ZP treatments at concentrations of 0.001% and 0.005% significantly reduced ROS levels by 25.4% ± 7.2 (*p* = 0.0035) and 29.3% ± 6.2 (*p* = 0.003), respectively, compared to the control + UD + UVA group. Moreover, when the baseline values of oxidative stress were subtracted for all samples, the results indicated that ZP treatments at 0.001% and 0.005% significantly protected the samples from UD +UVA-induced ROS levels by 27.3 ± 7.8% (*p* = 0.0036) and 31.5 ± 6.7% (*p* = 0.003), respectively, compared to the control + UD + UVA (Figure 7).

#### 3.2.3. Anti-Inflammatory Assessment against UD-Induced IL-6 and IL-1α Production

**Figure 8:**
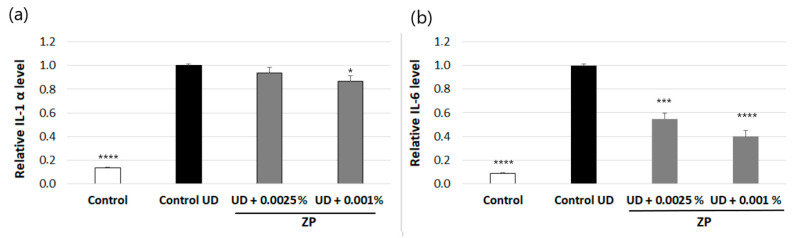
IL-1α (**a**) and IL-6 (**b**) protein levels in human keratinocytes after treatment with ZP at 0.0025% and 0.001% for 24 h and, normalized to UD-treated control. Data are presented as mean ± SEM of 4 technical replicates per condition. Asterisks indicate significant differences vs. control UD as follows: * *p*-value < 0.05, *** *p*-value < 0.001, **** *p*-value < 0.0001.

The results indicated that 24 h of incubation with UD at 0.4 mg/mL of human keratinocytes the increased protein levels of IL-1α and IL-6 by 86.3 ± 1% and 91.3 ± 6%, respectively, compared to the non-treated control (Figure 8a,b). 

The treatment of human keratinocytes with ZP at 0.0025% or 0.001% significantly decreased UD-induced IL-6 levels by 45 ± 6.1 or 59.9 ± 6.3%, respectively, compared to the UD-treated control (Figure 8b). Regarding IL-1α, in the conditions studied, only the dosage of 0.001% ZP slightly reduced the protein levels, by 13.5% (Figure 8a).

#### 3.2.4. Evaluation of AhR, Nrf2, and CYP1A1 Protein Levels in Human Keratinocytes

**Figure 9:**
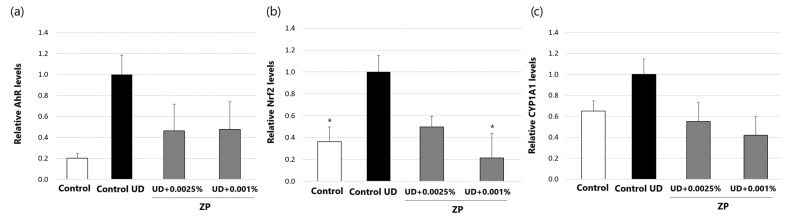
AhR (**a**) Nrf2 (**b**) and CYP1A1 (**c**) protein levels in human keratinocytes after treatment with ZP at 0.0025% and 0.001% for 24 h and normalized to UD-treated control. Data are presented as mean ± SEM of 4 technical replicates per condition. Asterisks indicate significant differences vs. control UD as follows: * *p*-value < 0.05.

The test results indicated that incubating human keratinocytes with UD at 0.4 mg/mL for 24 h increased protein levels of Nrf2 by 63.7% (Figure 9b) compared to the control group that was not treated with UD. On the other hand, the study also revealed a toxic response associated with the activation of AhR and CYP1A1 proteins. However, it is important to note that these changes were not statistically significant (Figure 9a,c), possibly due to the low protein content observed in the samples.

The UD-activated Nrf2 levels were significantly reduced after treatment with ZP at 0.001% (by 78.5 ± 22%) compared to the UD-treated control (Figure 9b). In contrast, although AhR reduction was observed, as well as CYP1A1 protein levels in UD-treated vs. UD/ZP-treated conditions, this reduction was not statistically significant (Figure 9a,c). 

### 3.3. In Vitro Analysis of the Anti-Pollution Capacity of ZP in Human Pulmonary Fibroblasts

#### 3.3.1. Antioxidant Assessment against UD-Induced Oxidative Stress

To assess the cytotoxicity of UD in human pulmonary fibroblasts (HPFs) and its ability to induce oxidative stress, cell viability analysis using the MTT assay was conducted. This analysis was performed in parallel to evaluate the effect of UD on cell mortality and to normalize the levels of reactive oxygen species (ROS) to the number of viable cells in each specific condition.

**Figure 10:**
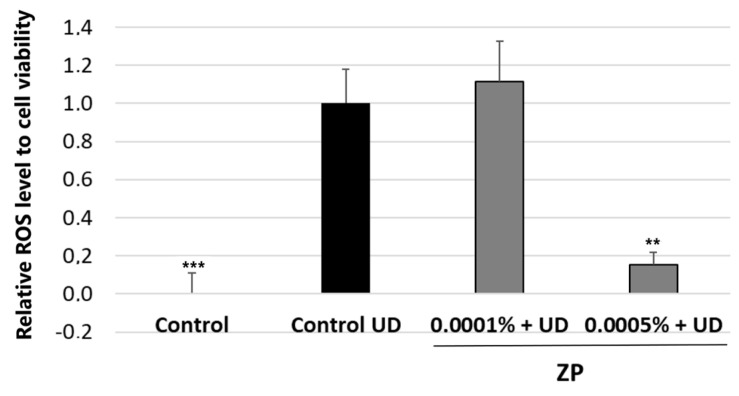
ROS accumulation relative to cell viability and normalized to control UD. Results were obtained in samples untreated and treated with ZP at 0.0001% and 0.0005%, and UD treatment for 24 h on HPF. Data are presented as mean ± SEM of 6 technical replicates per condition. Asterisks indicate significant differences vs. control UD as follows: ** *p* < 0.01 and *** *p* < 0.001.

The results of this study showed that treatment with UD at a concentration of 400 μg/mL significantly increased ROS levels by 90.3 ± 19.0% on HPFs, after 24 h of incubation, compared to the untreated control. To evaluate the protective capacity of ZP against UD-induced oxidative stress, the cells were also treated with the blend at a concentration of 0.0005% and 0.0001% for 24 h. Notably, the treatment with ZP at 0.0005% resulted in a significant decrease in ROS levels by 84.6 ± 18.9% (Figure 10). It is important to mention that these results were obtained after subtracting the baseline levels of oxidative stress from each condition.

#### 3.3.2. Study of mRNA Expression of BAX-2 and FAS Genes by RT-qPCR in HPF

In this assay, we assessed the capacity of ZP in counteracting the apoptotic effect induced by UD exposure in HPF, through analysis of BCL2-associated X (BAX2) and Fas cell surface death receptor (FAS1), using qPCR in vitro.

The Fas cell surface death receptor gene encodes the Fas receptor, which is a critical protein involved in the regulation of apoptosis in humans. The Fas receptor, also known as APO-1, is a transmembrane protein that belongs to the tumor necrosis factor (TNF) receptor superfamily and is part of the extrinsic apoptosis pathway. When the Fas ligand (FasL) binds to the Fas receptor, this leads to the formation of the intracellular death complex domain, which triggers a series of intracellular signaling events, such as activation of caspase 8, ultimately resulting in apoptosis [71]. 

In the same way, BAX2 is a gene that encodes a protein called Bax2, which belongs to the Bcl-2 family of proteins. Like the FAS gene and its receptor, the Bax2 protein plays a significant role in apoptosis. Bax2 is a pro-apoptotic protein involved in the intrinsic pathway of apoptosis. When cells receive apoptotic signals, Bax2 can be activated, leading to its insertion into the mitochondrial outer membrane. This process can lead to mitochondrial permeabilization, release of apoptotic factors, and, ultimately, cell death [72]. 

The expression and activity of Bax2 and FAS can be regulated by various cellular pathways and external factors, including the presence of stress signal ROS generated in response to pollutants [73].

**Figure 11:**
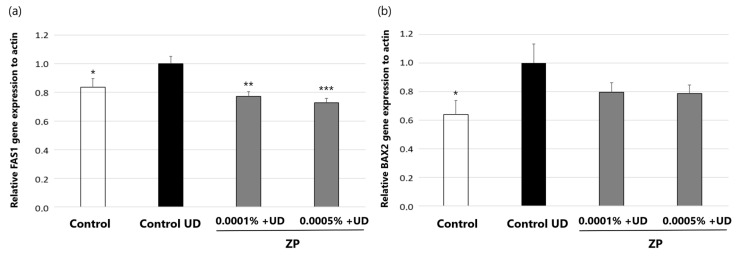
*FAS1* (**a**) and *BAX2* (**b**) gene expression relative to actin content and normalized to control UD after treating HPF with ZP at 0.0001% and 0.0005% concentrations for 24 h. Data are presented as mean ± SEM of 4 technical replicates per condition. Asterisks indicate significant differences vs. control UD as follows: * *p*-value < 0.05, ** *p*-value < 0.01, *** *p*-value < 0.001.

The results of this study, represented in Figure 11, showed that treatment of HPF cells with UD for 24 h increased the expression of apoptotic genes *BAX2* (38.7 ± 17.3%) and *FAS1* (16.8 ± 8.1%), evidencing the pro-apoptotic activity of UD. On the other hand, ZP at 0.0001% and 0.0005% significantly inhibited *FAS1* gene expression by 23.2 ± 6.2% and 27.7 ± 6.1%, respectively (Figure 11a). Also, although not statistically significant (*p* = 0.13), a trend was observed in the reduction in *BAX 2* expression when cells were treated with ZP at 0.0001% (−22.1 ± 15.0%) and 0.0005% (−22.8 ± 14.4%) (Figure 11b). These results suggest that the product may in part counteract the apoptotic effect originating from UD exposure in this cell model.

### 3.4. In Vitro Antioxidant Assessment against UD-Induced Oxidative Stress in Human Endothelial Cells (HUVECs)

**Figure 12:**
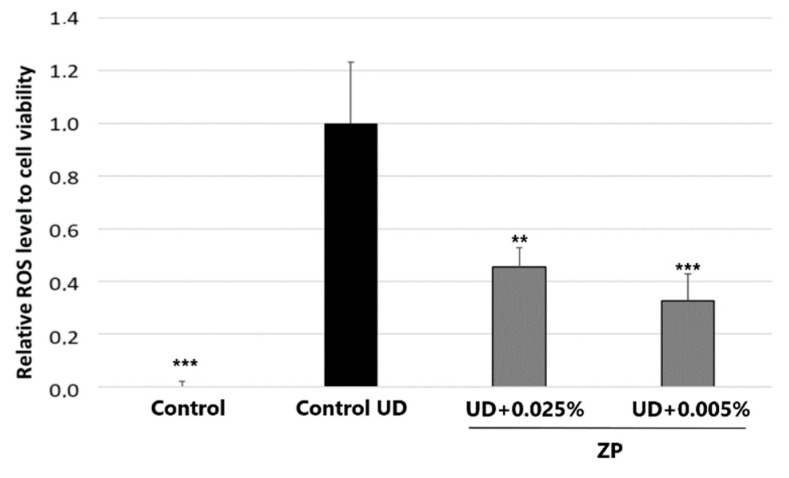
ROS accumulation relative to cell viability and normalized to control UD in HUVEC cells. Results were obtained in samples untreated and treated with ZP at 0.0001% and 0.0005%, and UD treatment for 48 h on HPF. Data are presented as mean ± SEM of 6 technical replicates per condition. Asterisks indicate significant differences vs. control UD as follows: ** *p* < 0.01 and *** *p* < 0.001.

The HPF cell model was used to evaluate the effect of UD on cell mortality and to normalize the levels of reactive oxygen species (ROS) to the number of viable cells in each specific condition. Cell viability was assessed using the MTT assay. The results indicated that treatment with UD at 400 μg/mL on HPFs and on HUVECs significantly induced ROS levels by 866.6 ± 201.9%, compared to the untreated control, after 24 h of incubation. As can be seen in Figure 12, when HUVECs were treated with ZP at 0.0005% and 0.001%, there was a significant reduction in UD-induced oxidative stress levels by 54.7 ± 24.4% and 67.4 ± 25.3%, respectively. These results were obtained after the subtraction of the basal values of oxidative stress from each of the conditions.

### 3.5. Heartbeat Quantification in Medaka Embryos Exposed to Urban Dust

**Figure 13:**
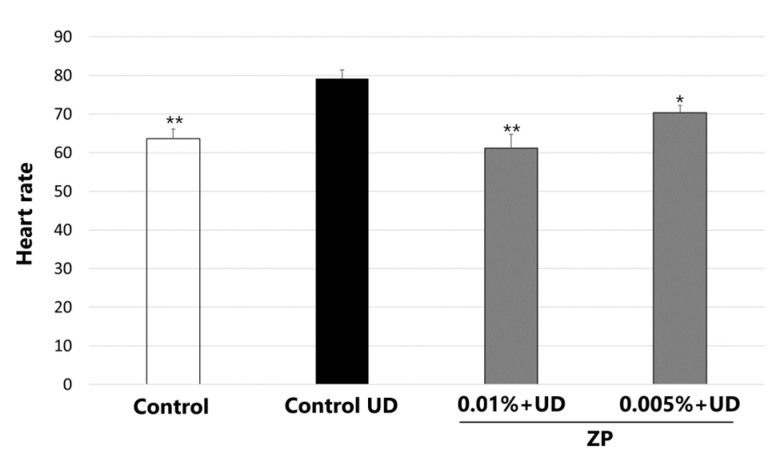
Results showing ZP effect on heart rate of medaka embryos after UD exposure. Bar graphs represent heart rate in samples non treated or treated with ZP at 0.005% and 0.01% and treatment with UD at 250 μg/mL for 48 h. Data are presented as mean ± SEM of 5 technical replicates per condition. Asterisks indicate significant differences vs. control UD as follows: * *p* < 0.05 and ** *p* < 0.01.

In this study, we assessed the efficacy of ZP in counteracting the UD impact on the heart rate. The results indicated that treatment with UD at 250 μg/mL significantly increased the heart rate by 24.5 ± 5.3% compared to the untreated control after 48 h of incubation, and the medaka´s heart rate went from 63.9 beats per minute (bpm) to 79.2 bpm (Figure 13). When the medaka embryos were treated with ZP at 0.01%, a significant drop in heart rate was observed (22.4 ± 5.6%) compared to control values, counteracting the effect produced by UD. Treatment at 0.005% decreased the heart rate (11.0 ± 3.7%), even though the results were not statistically significant (*p* > 0.05), compared to the control + UD (Figure 13). Videos S1–S3 of medaka heart rate are included in the supplementary section. 

## 4. Discussion

It is well-known that prolonged exposure to air pollution significantly contributes to an increase in non-communicable diseases such as allergies and cardiovascular, respiratory and metabolic diseases. While the exact mechanism underlying air-pollution-induced damage is unknown, a complex interplay between oxidative stress, inflammation, and various metabolic pathways has been identified in different studies. Studies have shown that exposure to particulate matter components (inorganic and organics, mostly metals and PAHs) enhances ROS formation and alters mitochondrial function, which may lead to inflammation. The disturbance of redox homeostasis alters the activation of redox-sensitive signaling pathways such as Nrf2. Additionally, PAHs activate the AhR pathway, further contributing to the physiopathological inflammatory effects of PM [74]. 

As air pollution continues to worsen, new approaches to preventing its harmful effects are needed. Food and dietary supplements that incorporate botanicals with components known for their antioxidant and anti-inflammatory properties emerge as a potential strategy to mitigate the detrimental effects of environmental pollution.

Previously, in a clinical trial, we proved the skin anti-aging and antioxidant effects of Zeropollution^®^ (ZP) in a population exposed to high levels of environmental pollution, particularly PM10 and PM2.5. We showed that subjects taking 250 mg daily of ZP for 3 months presented a lower skin lipid peroxidation, while their antioxidant status, measured by FRAP in the saliva, was improved compared to the placebo group [42]. 

ZP includes active compounds with potent antioxidant properties, making it a promising candidate for mitigating the detrimental effects of environmental pollution. These active ingredients include hydroxytyrosol, verbascoside, oleuropein, rosemary diterpenes, and quercetin. 

The antioxidant and anti-inflammatory effects of olive polyphenols, such as oleuropein and hydroxytyrosol, have been extensively confirmed in the scientific literature [50,75,76]. Also, there is some evidence to suggest that they may offer protection against pollution. A study conducted on rats demonstrated that hydroxytyrosol had a significant impact on mitigating PM2.5-induced insulin resistance by inhibiting the activation of NF-κB, which is triggered by oxidative stress [77]. Furthermore, in other research, it was shown that a low dose of hydroxytyrosol was effective in decreasing the adverse effects of oxidative stress induced by side-stream smoke in rats [49]. 

Also, verbascoside, a hydrophilic caffeoyl phenylethanoid glycoside present in *Lippia citriodora*, offers both antioxidant and anti-inflammatory benefits [51,78,79]. In lung cells, it exerts anti-inflammatory and antioxidant effects by inhibiting the activity of inflammatory mediators and the NF-κB pathway, while also enhancing the activity of antioxidant enzymes, thereby ameliorating cell injury [80]. More recently, it has been proven that verbascoside offers protection to pulmonary cells against paraquat-induced toxicity through the reduction in ROS and the production of inflammatory markers. Additionally, it suppresses the increased activity of NF-κB and caspase-3, inhibits the formation of 8-OHdG, and ultimately enhances cell viability [81]. 

*Rosmarinus officinalis* is an aromatic plant with a rich history in herbal remedies due to its diverse biological activities including antioxidant, antimicrobial, anti-inflammatory [82,83] and skin rejuvenation properties [84]. Studies have shown that rosemary extract exhibits strong antioxidant properties linked to its polyphenol content, notably carnosic acid [53,82]. A recent study that assessed the impact of spray-dried algae-rosemary particles (RSPs) on pollution-induced damage using human biopsies exposed to diesel engine exhaust showed that RSPs effectively reduced inflammatory responses in cutaneous tissue, lowering 4-hydroxynonenal protein adducts and active MMP-9 levels, indicating its potential to counteract pollution-induced skin aging/damage [85].

Finally, *Sophora japonica* holds a well-established position in Chinese herbal traditional medicine, with a multitude of documented biological activities, including antioxidant, antibacterial, anti-allergic, and anti-inflammatory properties [86]. Moreover, research has explored the anti-pollution benefits associated with Sophora japonica and quercetin mainly due to their antioxidant and anti-inflammatory properties [38,87,88].

The purpose of this study was to investigate the protective effects of the dietary supplement ZP against pollution-induced damage in cutaneous and cardiopulmonary systems, as well as in endothelial cells and medaka fish (*Oryzias latipes*) embryos. 

Oxidative stress, inflammation, and disruption of metabolic processes in the skin are considered the primary causes of pollution-derived skin disorders [4,17,20]. Moreover, when the skin is also exposed to UV, this toxic stress is increased [18]. 

The results obtained in keratinocytes and skin explants collectively suggest that ZP could possess significant benefits for skin exposed to pollutants. This study revealed that ZP, through its polyphenolic components, demonstrated a significant capacity to mitigate lipid peroxidation and reduce inflammation (IL-1α reduction) in human skin explants exposed to a mixture of pollutants. Furthermore, modulation of the expression levels of AhR, MT-1H, and Nrf2 was observed. Similar results also were found in human keratinocytes exposed to PM-UD. In this in vitro model, ZP also showed a significant antioxidant capacity by reducing levels of reactive oxygen species (ROS) induced by UD alone or in combination with UVA radiation. Additionally, a decrease in the expression of inflammatory markers such as IL-6 and IL-1α in response to ZP treatment was observed. These results are consistent with previous research highlighting the anti-inflammatory and antioxidant potential of phenolic compounds in skin cells [51,52,84,89]. Moreover, the negative modulation of AhR and Nrf2 by ZP suggests a potential interference in key signaling pathways related to oxidative stress. Different studies have proven the capacity of various polyphenols present in the formula to inhibit the AhR activation in response to toxic substances and UV radiation. This is observed with quercetin [90,91], verbascoside [81], and the diterpene carnosol [92].

It is well-understood that pollution contributes to extrinsic skin aging including pigment irregularities, nasolabial folds, and wrinkles. It is also an aggravating factor in several inflammatory or allergic skin conditions [20]. With the increasing need for environmental protection of the skin beyond sunscreens and an increasing consumer desire for plant-based cosmetics, either as oral supplements or topical products, plant derived anti-pollution strategies are being given increasing attention. By reducing oxidative stress, inflammation, lipid peroxidation and AhR overactivation, ZP provides a comprehensive protective mechanism for the skin exposed to environmental pollutants. The findings align with the observations made in the clinical study involving this ingredient, where we demonstrated that the intake of ZP for 12 weeks significantly improved systemic and skin oxidative status, strengthened the skin barrier, improved skin moisturization, regulated sebum secretion and provided anti-aging skin benefits versus the placebo group in women exposed to a daily high levels of environmental pollution [42].

In addition, we studied whether ZP could have any benefits on the cardiopulmonary system. ZP demonstrated a significant ability to reduce oxidative stress induced by UD in human lung fibroblasts. This protective effect was evidenced by a significant decrease in ROS. Additionally, an attenuation of the expression of the apoptotic genes FAS and BAX2 was observed, indicating a possible role in preventing apoptosis induced by PM pollutants. These results are in line with previous research indicating the antioxidant properties, and antiapoptotic properties of at least some of the polyphenolic compounds present in ZP, in lung cells exposed to different aggressions [80,81,93]. 

Human umbilical vein endothelial cells (HUVECs), a subset of human vascular endothelial cells, are frequently used as a model for cardiovascular research due to their key role in regulating blood pressure, atherogenesis, and thrombosis. There is recent evidence that supports the relationship between PM air pollution and endothelial dysfunction [29,94,95]. Oxidative stress can lead to vascular aging and beneficial antioxidant-based agents such as N-acetylcysteine and vitamin C can improve aging-related endothelial cells’ functioning [96,97]. In this study, ZP was proven to contribute to protecting endothelial cells from oxidative stress induced by UD by reducing the ROS production. Furthermore, we observed that ZP treatment exerts a beneficial influence on cardiovascular health, counteracting the adverse effects of UD on the heart rate of medaka embryos exposed to UD. Although the precise mechanism of action remains unknown, it is possible that ZP’s ability to inhibit the response of the AhR receptor to pollution and its ability to reduce the generation of ROS might play a pivotal role. Several studies suggest that AhR dysregulation mediates the cardiac developmental toxicity of environmental chemicals [98,99], while excessive ROS at the late stage can inhibit cardiac development via oxidative stress and apoptosis [100]. Also, recent studies reported that PM significantly increased the heart malformation rates in zebrafish embryos, while the inhibition of AhR signaling or ROS generation could reduce PM-induced heart defects [101,102]. However, additional studies would be needed to elucidate the exact mechanism.

Prior research has consistently highlighted the role of oxidative stress, inflammation, and cellular damage in pollution-induced health issues, corroborating the mechanisms addressed in our study. However, it is essential to acknowledge the limitations of our work. While our results are promising, this study primarily relied on in vitro and ex vivo models. Further research involving in vivo models and clinical trials is warranted to fully understand the extent of ZP’s protective effects in real-world scenarios. Additionally, variations in pollution levels and compositions may influence ZP’s efficacy, which requires further investigation. Finally, the contribution of each individual extract was not studied in these models, which covered only the efficacy of the combination of the four herbal extracts. 

Future research should explore the precise molecular pathways by which ZP exerts its protective effects and delve deeper into its mechanisms of action and how exactly the individual extracts in ZP contribute. Moreover, large-scale clinical trials are necessary to assess the supplement’s efficacy in diverse populations exposed to varying levels of air pollution. These trials should also consider long-term outcomes to determine whether ZP can serve as a sustainable strategy for preventing pollution-induced health issues.

## 5. Conclusions

ZP through its antioxidant, anti-inflammatory and modulatory aryl hydrocarbon receptor properties, among others, offers a promising solution to reduce the harmful health effects of air pollution on different body systems: the skin, respiratory system and cardiovascular system. However, further investigations are required to translate these findings into practical and effective preventive measures.

## 6. Patents

Monteloeder SL. Composición De Extractos Vegetales Con Flavonoides Para Paliar Los Múltiples Efectos De La Contaminación Del Aire Sobre La Piel. WO/2019/211501, 7 November 2019.

## Figures and Tables

**Table 1:** Pollution treatment plan of skin explants.

Batch	Treatment	Pollutants Exposure	Number of Explants	Sampling Time
*T*0	Untreated control	−	3	Day 0
*T*	Untreated control	−	4	Day 3
*ZP*	ZP treatment	−	4	Day 3
*P*	Pollution treatment	+	4	Day 3
*PZP*	Pollution + ZP	+	4	Day 3

− non exposure, + exposure. T0 (untreated control skin explants at day 0); T (untreated control skin explants at day 3); ZP (skin explants treated with ZP); P (skin explants exposed to pollutants); ZP (skin explants exposed to pollutants and treated with ZP).

## Data Availability

Data are contained within the article and supplementary materials.

## Supplementary Materials

The following supporting information can be downloaded at: https://www.mdpi.com/article/10.3390/cimb46020099/s1, Figure S1: Flow Chart ZP; Figure S2: Representative high-performance liquid chromatography (HPLC) chromatogram of the compounds identified in the blend; Table S1: Table of Compounds Identified by HPLC-MS; Table S2: Composition of heavy metals and hydrocarbons solution; Table S3: Compositional analysis from Urban Dust (heavy metals); Videos S1–S3 of medaka heart rate.
