# Supplementary material for: Protective Potential of a Botanical-Based Supplement Ingredient against the Impact of Environmental Pollution on Cutaneous and Cardiopulmonary Systems: Preclinical Study
Source: Curr Issues Mol Biol. 2024 Feb 15;46(2):1530–55. doi: 10.3390/cimb46020099 (PMC10887869; doi:10.3390/cimb46020099)
Supplement: Supplementary file 1 [file cimb-46-00099-s001.zip › cimb-2855645-supplementary.pdf]

Protective Potential of a Botanical-Based Supplement Ingredient against the Impact of Environmental Pollution on Cutaneous and Cardiopulmonary Systems: Preclinical Study

Table S1: Table of Compounds Identified by HPLC-MS.

Compounds Identified	RT	Mass	Formula	Tgt Mass	Diff (ppm)
Hydroxytyrosol	9.977	154.0626	C8 H10 O3	154.063	-2.76
Chlorogenic acid	15.126	354.096	C16 H18 O9	354.0951	2.52
Caffeic acid	17.179	180.0426	C9 H8 O4	180.0423	1.66
Tyrosol	17.307	138.0693	C8 H10 O2	138.0681	9
Verbascoside	23.338	624.2078	C29 H36 O15	624.2054	3.85
Rutin	24.14	610.1555	C27 H30 O16	610.1534	3.52
Luteolin-7-O-glucoside	24.99	448.1019	C21 H20 O11	448.1006	3.04
Apigenin-7-O-glucoside	25.984	432.1066	C21 H20 O10	432.1056	2.19
Apigenin-7-O-glucuronide	26.049	446.0884	C21 H18 O11	446.0849	7.82
Rosmarinic acid	28.134	360.0856	C18 H16 O8	360.0845	3.08
Oleuropein	29.048	540.186	C25 H32 O13	540.1843	3.25
Luteolin-7-O-glucuronide	29.866	462.0817	C21 H18 O12	462.0798	4.08
Quercetin	34.518	302.0439	C15 H10 O7	302.0427	4.23
Carnosol	54.407	330.1843	C20 H26 O4	330.1831	3.6
Carnosic acid	57.615	332.1969	C20 H28 O4	332.1988	-5.5
12-Methyl carnosic acid	59.812	346.2152	C21 H30 O4	346.2144	8

RT: retention Time; Mass: molecular weight; Tgt Mass: target mass; Diff (ppm): difference between the Mass detected and the target mass.

HPLC-MS method used to identify active compounds in the four botanical blend: HPLC/MS system consisted of HPLC Agilent 1200/TOF AGILENT 6220 Mass Spectrometer (Agilent Technologies Inc., Palo Alto, CA, USA) controlled by the Chemstation software. A Teknokroma Mediterranea sea RP-18, 5 µm, 250 x 0.46 cm column was used for analytical purposes. The mobile phases consisted in acetic acid 2.5% (A) and acetonitrile (B). The following multi-step linear gradient was applied: 0 min, 5% B; 22 min, 25% B; 45 min, 50% B; 55 min, 20% B; 66 min, 5% B; 70 min, 5% B. The flow rate was 0.8 mL/min, and the column temperature was 30ºC. The injection volume in the HPLC system was 20 µl. The ESI source was operated in negative mode to generate [MH] ions using the following conditions: desolvation temperature at 350ºC and vaporizer temperature at 400ºC; dry gas (nitrogen) and nebulizer were set at 12 L/min and 60 psi, respectively. VCap voltage was set at 3000 V, fragmentor at 180 V, skimmer at 65 V and Octopole RF peak at 250 V. The MS data were acquired as full scan mass spectra at 50–1200 m/z by using 50 ms for collection of the ions in the trap.

Table S2. Composition of heavy metals and hydrocarbons solution

Final concentrations applied on the explants of each compound of ICP multielement standard V Certi Pur ® solution (Merck ; reference 1.10714.0500 ; batch HC309202) supplemented with hydrocarbons and diesel particles:

**Heavy metals**	
Al	0.01 mg/mL
AS	0.01 mg/mL
B	0.001 mg/mL
Ba	0.001 mg/mL
Be	0.0005 mg/mL
Ca	0.005 mg/mL
Cd	0.001 mg/mL
Cr	0.001 mg/mL
Cu	0.001 mg/mL
Fe	0.001 mg/mL
Hg	0.0025 mg/mL
K	0.0495 mg/mL
Li	0.001 mg/mL
Mg	0.0005 mg/mL
Mn	0.0005 mg/mL
Na	0.01 mg/mL
Ni	0.0025 mg/mL
P	0 .005 mg/mLg
Pb	0.01 mg/mL
Sc	0.0005mg/mL
Sa	0.01 mg/mL
Sr	0.000 5mg/mL
Te	0.01 mg/mL
Ti	0.001 mg/mL
Y	0.0005 mg/mL
Zn	0.001 mg/mL
**Hydrocarbons**	
Benzene	1µL/ml
Xylene	1µL/mL
Toluene	1 µl/ml
**Diesel particles (DP):**	0.1 mg/mL in the pollutant mixture solution

Table S3: Compositional analysis from Urban Dust (heavy metals)

* Analytic report using Atomic Spectrometry

Element	Units	Urban Dust	Element	Units	Urban Dust
Ca	mg/kg	131.458,50	Gd	mg/kg	5,55
Al	mg/kg	16.072,00	Co	mg/kg	4,74
Mg	mg/kg	15.374,50	I	mg/kg	4,73
Fe	mg/kg	14.995,00	Pr	mg/kg	3,94
Si	mg/kg	9.350,50	Nb	mg/kg	3,71
K	mg/kg	7.186,00	Bi	mg/kg	2,39
Na	mg/kg	6.161,00	Cs	mg/kg	2,13
P	mg/kg	2.332,50	Sm	mg/kg	2,07
Zn	mg/kg	2.063,00	Dy	mg/kg	1,81
Ti	mg/kg	973,50	Ag	µg/Kg	1.519,00
Ba	mg/kg	666,00	Yb	µg/Kg	1.370,50
Cu	mg/kg	339,50	Hf	µg/Kg	1.066,50
Mn	mg/kg	256,00	Er	µg/Kg	958,50
Sr	mg/kg	241,50	U	µg/Kg	877,50
Ce	mg/kg	194,70	Eu	µg/Kg	765,50
Pb	mg/Kg	130,80	Tl	µg/Kg	598,00
Cr	mg/kg	81,95	Hg	µg/Kg	460,50
Sn	mg/kg	64,40	Ho	µg/Kg	417,00
V	mg/kg	43,65	Au	µg/Kg	363,50
Br	mg/kg	42,05	In	µg/Kg	229,50
Ni	mg/kg	41,15	Tb	µg/Kg	205,00
Rb	mg/kg	28,80	Tm	µg/Kg	147,50
Sb	mg/kg	26,20	Pd	µg/Kg	147,00
Zr	mg/kg	26,00	Cd	µg/Kg	137,50
La	mg/kg	19,10	Ta	µg/Kg	89,50
Nd	mg/kg	17,55	Lu	µg/Kg	87,50
B	mg/kg	16,90	Pt	µg/Kg	79,50
As	mg/kg	14,88			
Mo	mg/kg	14,15			
W	mg/kg	10,71			
Y	mg/kg	10,69			
Th	mg/kg	5,96			

Figure S1: Flow Chart ZP

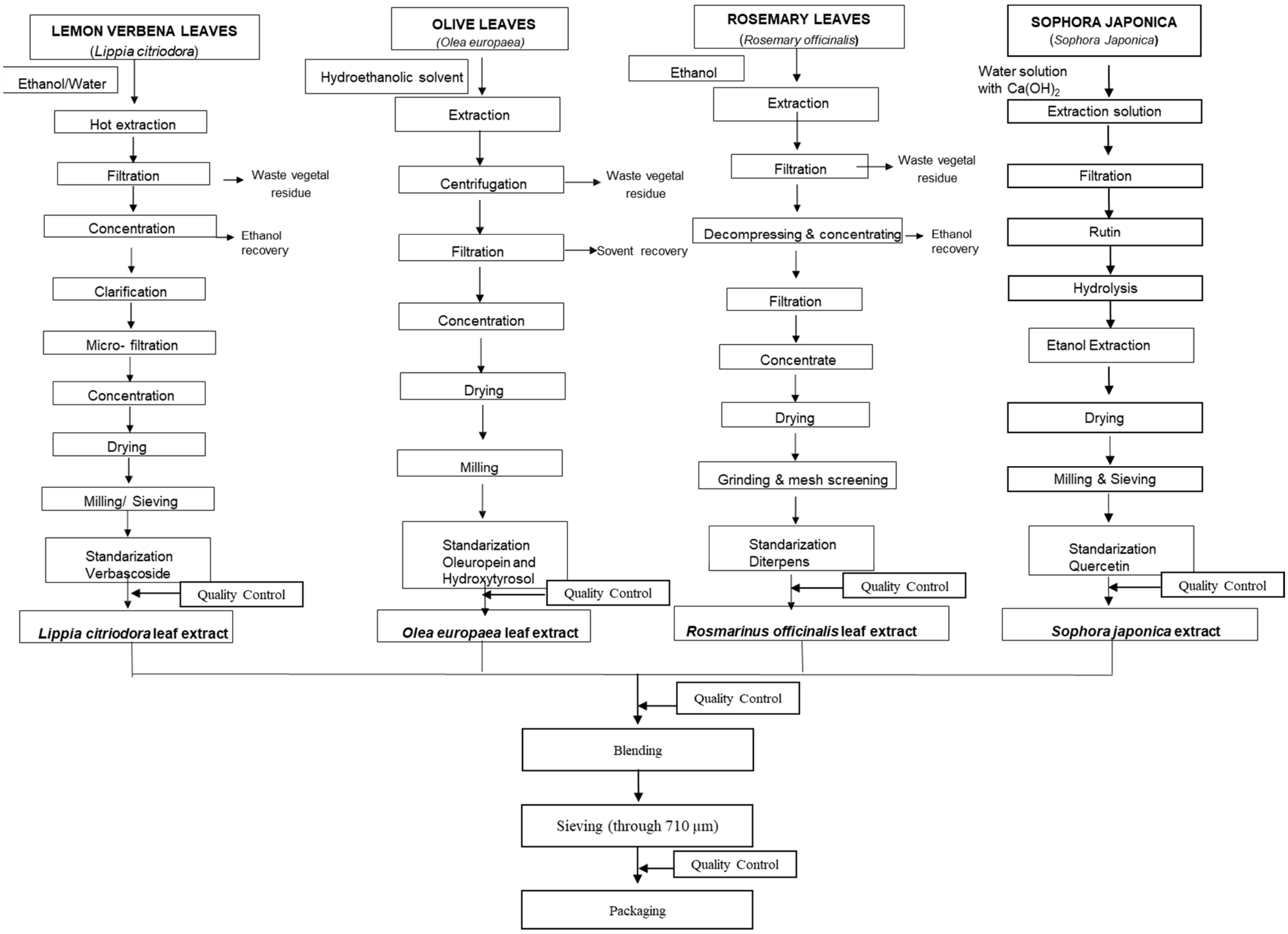

Figure S2: Representative high-performance liquid chromatography (HPLC) chromatogram of the compounds identified in the blend.

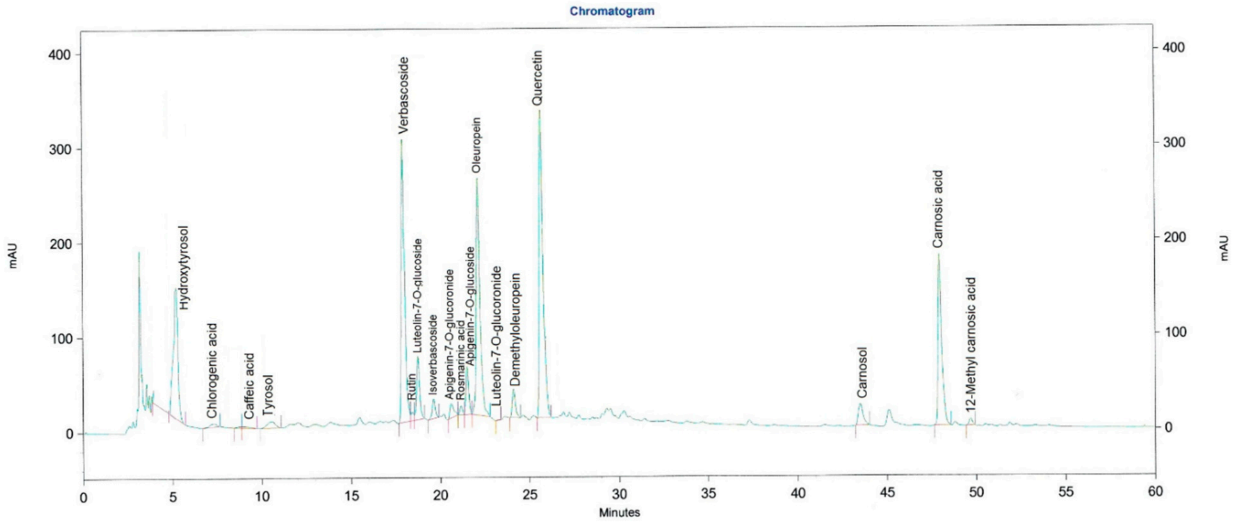

Method used to identify and quantify main active compounds by HPLC: The composition of the combination of extracts was identified and quantified by using an HPLC with Array Photodiode (HPLC-DAD), VWR Hitachi Elite, comparing the retention time and UV spectra of the peaks in the samples with those of authentic standards or data

reported in the literature. A BDS Hypersil C18 (5μm, 250×4 mm) column was used for analytical purposes. The separation of the compounds was carried out at 30ºC with a gradient elution program at a flow rate of 1 ml min^{-1}. The mobile phases consisted in acetic acid 2.5% (A) and acetonitrile (B). The following multi-step linear gradient was applied: 0 min, 5% B; 20 min, 25% B; 40 min, 50% B; 50 min, 20% B; 60 min, 5% B. The injection volume in the HPLC system was 20 μl. The UV–vis detection was performed in the 190–450 nm range. Different wavelengths were set to quantify the main active compounds: 280nm for Hydroxytyrosol and Oleuropein, 330nm for verbascoside, 370nm for quercetin and 230 nm for carnosic acid, carnosol y 12-methyl carnosic acid (diterpens). Each main active was identified by comparison of the UV-Vis spectrum and retention time with the correspondence of the Reference Standard (RS) and its content (dry basis) measured by area comparison of the sample peak versus standard peak.
